# Caries dental en dos poblaciones incas. un estudio transversal

**DOI:** 10.21142/2523-2754-1202-2024-195

**Published:** 2024-06-27

**Authors:** Darwin Ortiz de Orué Ninantay, Ebingen Villavicencio Caparó, María del Carmen Peña Alegre

**Affiliations:** 1 Departamento Académico de Odontología de la Universidad Nacional de San Antonio Abad del Cusco. Cusco, Perú. Docente de la Escuela Profesional de Estomatología de la Universidad Tecnológica de los Andes. Abancay, Perú. darwin.ortizdeorue@unsaac.edu.pe Universidad Tecnológica de los Andes Departamento Académico de Odontología Universidad Nacional de San Antonio Abad del Cusco Cusco Peru darwin.ortizdeorue@unsaac.edu.pe; 2 Departamento Académico de Odontología Social de la Universidad Peruana Cayetano Heredia. Lima, Perú. Docente de la carrera de Odontología de la Universidad Católica de Cuenca. Cuenca, Ecuador. evillavicencioc@ucacue.edu.ec Universidad Católica de Cuenca Departamento Académico de Odontología Social Universidad Peruana Cayetano Heredia. Lima Ecuador evillavicencioc@ucacue.edu.ec; 3 Departamento Académico de Odontología de la Universidad Nacional de San Antonio Abad del Cusco. Cusco, Perú. maria.pena@unsaac.edu.pe Universidad Nacional San Antonio Abad del Cusco Departamento Académico de Odontología Universidad Nacional de San Antonio Abad del Cusco Cusco Peru maria.pena@unsaac.edu.pe

**Keywords:** paleopatología, odontología, salud bucal, caries dental, paleopathology, dentistry, oral health, dental caries

## Abstract

**Objetivo:**

Comparar : frecuencia, severidad y localización de la caries dental entre poblaciones arqueológicas de las muestras Sacsayhuamán y Machupicchu.

**Material y métodos:**

Se trató de un estudio retrospectivo, transversal y observacional. La variable caries dental fue observada directamente en las muestras Sacsayhuamán y Machupicchu de la Dirección de Cultura Cusco. Se analizaron 39 individuos de la muestra Sacsayhuamán, con 566 piezas dentarias, y 49 individuos de la muestra Machupicchu, con 467 piezas dentarias. La unidad de análisis fue la pieza dentaria, mientras que la variable fue analizada estadísticamente por frecuencias y proporciones, y las diferencias, mediante la prueba de chi-cuadrado.

**Resultados:**

En la muestra Sacsayhuamán, las piezas con caries tuvieron una frecuencia del 31,8%, mientras que en la muestra Machupicchu alcanzaron una frecuencia del 23,6%. Respecto de la localización, en la muestra Sacsayhuamán se obtuvo las siguientes frecuencias: caries oclusal: 44,38%, caries coronaria: 3,37%, caries en la línea amelocementaria: 32,58% y caries radicular: 19,66%; mientras que en la muestra Machupicchu, caries oclusal: 60,91%, caries coronaria: 3,62%, caries en la línea amelocementaria: 24,55% y caries radicular: 12,91%. Con relación a la severidad, encontramos las siguientes frecuencias: en la muestra Sacsayhuamán, caries esmalte/cemento: 52,81%, caries de dentina: 33,71%, caries de afectación pulpar: 13,14%; por su parte, en la muestra Machupicchu, caries esmalte/cemento: 49,09%, caries de dentina: 27,27% y caries de afectación pulpar: 23,64%.

**Conclusiones:**

Al comparar las muestras, se encontraron diferencias con relación a la frecuencia de caries dental y respecto de su localización, mas no se hallaron diferencias en cuanto a su severidad.

## Abstract (en)

**Objective:**

Compare: frequency, severity and location of dental caries between archaeological populations of the Sacsayhuamán and Machupicchu samples.

**Material and Methods:**

This was a retrospective, cross-sectional and observational study. The variable dental caries was observed directly in the Sacsayhuamán and Machupicchu samples from the Cusco culture directorate. 39 individuals from the Sacsayhuamán sample with 566 teeth and 49 individuals from the Machupicchu sample with 467 teeth were analyzed. The unit of analysis was the tooth. The variable was statistically analyzed by frequencies and proportions and the differences were analyzed using the Chi test. -square.

**Results:**

In the Sacsayhuamán sample, pieces with cavities had a frequency of 31.8%, while in the Machupicchu sample they reached a frequency of 23,6%. Regarding the location: in the Sacsayhuamán sample: The following frequencies were obtained: occlusal caries 44,38%, coronary caries 3,37%, caries in the amelocemental line 32,58%, root caries 19,66%; while in the Machupicchu sample the following were obtained: occlusal caries in 60,91%, coronary caries in 3,62%, caries in the amelocemental line in 24,55%, root caries in 12,91%. Regarding severity, we found the following frequencies: in the Sacsayhuamán sample: enamel/cementum caries in 52,81%, dentin caries 33,71%, pulp involvement caries 13,14%, while in the Machupicchu sample: enamel/cementum caries 49,09%, cavities of dentin in (27,27%), caries involving pulp involvement 23,64%.

**Conclusions:**

When comparing the samples, differences were found with respect to the frequency of dental caries and with respect to the location, but no differences were found with respect to the severity.

## INTRODUCCIÓN

La paleopatología es una disciplina histórico-médica ^1^. Los restos humanos (momias y esqueletos) de culturas anteriores representan un enorme bioarchivo adecuado para la reconstrucción de las condiciones de vida y las enfermedades de poblaciones pasadas, incluidas pruebas de enfermedades infecciosas y traumas violentos ^2^. Se trata de una ciencia pluridisciplinar, razón por la cual también se interesan por ella los médicos, odontólogos, biólogos, paleontólogos, arqueólogos y todas aquellas personas preocupadas por la forma de vida en la prehistoria y en el mundo antiguo ^3^^,^^4^. Por ello, podemos definir a la paleopatología como la ciencia que estudia las enfermedades que pueden demostrarse en restos humanos y animales de la antigüedad ^5^. La paleodontología o paleoestomatología es una parte de la paleopatología, la cual se centra en el estudio de las estructuras, funciones y enfermedades del aparato o sistema estomatognático, a partir de restos humanos. Por lo tanto, se trata de una ciencia de carácter retrospectivo ^1^^,^^3^^,^^4^.

La caries dental es una condición influenciada por la presencia del biofilm en la boca, que a su vez se ve afectada por la dieta. Se trata de una enfermedad dinámica y compleja, causada por múltiples factores, no contagiosa, que conduce a la pérdida gradual de la porción mineral de los dientes. Este proceso, influenciado por aspectos biológicos, comportamentales, psicosociales y del entorno, resulta en la formación de lesiones cariosas ^6^. Es la enfermedad crónica dental más frecuente que afecta a la raza humana, por lo que es considerada una pandemia mundial, ya que afecta aproximadamente al 60-90% de los niños y casi el 100% de los adultos en todo el mundo ^7^.

En cuanto a Sudamérica, específicamente en el Perú, los pobladores del Ande eran menos susceptibles a la caries dental en comparación con los pobladores de la costa y la selva ^8^; de allí se puede concluir que cuanto más elevado (sobre el nivel del mar) era el asentamiento de una población, menos susceptible era a la caries y cuanto más bajaba su ubicación hacia la costa era más susceptible a la enfermedad. En la costa también existieron diferencias en cuanto a la afectación, siendo más afectados los pobladores de la costa centro y sur, según Borja *et al*. ^8^.

A raíz de diversas excavaciones arqueológicas realizadas en la región del Cusco, se dispone de restos óseos humanos catalogados como colecciones osteológicas, que se encuentran en poder de la Dirección Regional de Cultura Cusco. Estos corresponden a periodos diversos, como preínca, inca y colonial, y en ellos se pueden observar aparentes signos de enfermedad o patologías bucales limitadas al diente y las estructuras de soporte ^9^^,^^10^. Por ello, el presente estudio toma dos colecciones osteológicas de excavaciones del parque arqueológico de Sacsayhuamán y del parque arqueológico de Machupicchu, ambas -según estudios previos realizados por el personal del Gabinete de Antropología Física de la mencionada Dirección- pertenecientes al periodo de ocupación inca Horizonte tardío (1440-1532). El objeto de análisis fue la caries dental, con el fin de encontrar las diferencias en su frecuencia, severidad y localización, y tratar de explicar las posibles causas de dichas diferencias.

Por todo lo expuesto, la presente investigación tuvo como objetivo comparar la frecuencia, severidad y localización de la caries dental en población arqueológica de las muestras incas Sacsayhuamán y Machupicchu.

## MATERIALES Y MÉTODOS

El presente estudio tuvo un diseño descriptivo, retrospectivo y transversal, mediante observación macroscópica de las piezas dentales y las lesiones de caries. Las consideraciones éticas incluyeron la conservación y correcta manipulación del material bioarqueológico, para lo cual se obtuvo la aprobación de la institución que custodia las muestras; asimismo, todo el proceso de recolección de la información fue supervisado por el personal del Gabinete de Antropología Física de la Dirección Regional de Cultura Cusco. La población de estudio incluyó restos óseos humanos pertenecientes a individuos de las muestras arqueológicas incas de Machupicchu y Sacsayhuamán, distribuidos de la siguiente forma:

Grupo 1: restos óseos humanos de 39 individuos de la colección osteológica de Sacsayhuamán, exhumados de los sitios de Suchuna, Chincana Grande y Muyucmarca, dentro del parque arqueológico de Sacsayhuamán. Se evaluaron 566 dientes (maxilares y mandibulares) pertenecientes a estos individuos.

Grupo 2: restos óseos humanos de 49 individuos de la colección osteológica de Machupicchu, exhumados de los sitios de Salapunku y Patallacta, dentro del parque arqueológico de Machupicchu. Se evaluaron 467 dientes (maxilares y mandibulares) pertenecientes a estos individuos.

La selección se realizó por conveniencia, pues se eligieron las muestras que mejor estado de conservación presentaban. Ambas corresponden al periodo de ocupación inca (1536 aprox.) ^11^^,^^12^.

Las piezas dentarias fueron agrupadas en 4 grupos: incisivos, caninos, premolares y molares. Se utilizaron todas las piezas dentarias halladas, por ello el muestreo es por conveniencia, pues se usó todo el material a disposición y que cumplía el requisito previo de un buen estado de conservación, previamente determinado por estudios anteriores del personal de la institución.

Se incluyeron restos óseos humanos de individuos con determinación de sexo y edad provenientes de entierros bien contextualizados, es decir, certificados por un antropólogo de la Dirección Regional de Cultura Cusco, con dentición permanente, al menos 10 piezas dentarias bien conservadas y un maxilar por persona.

Se excluyeron los restos óseos humanos de individuos con dentición decidua o mixta, menores de 18 años, dientes sueltos de dudosa procedencia o en la condición de “no valorables”.

Se evaluó la caries dental con la técnica basada en el protocolo propuesto por Chímenos, Safont, Alesan, Alfonso y Malgosa ^3^^,^^4^. La caries dental, en cuanto a su localización, fue catalogada de la siguiente manera:

0: Estado no valorable. El mal estado o la ausencia de conservación del diente, en particular, no ofrece ninguna información en este caso.

1: Caries ausente. No se observa ninguna lesión cariosa en el diente estudiado.

2: Caries oclusal. Se observa(n) una o más lesiones cariosas, iniciada(s) en la cara oclusal del diente estudiado.

3: Caries coronal (coronaria). Lesión cariosa iniciada en cualquier cara de la corona que no sea la oclusal.

4: Caries en la línea amelocementaria (LAC). Lesión cariosa iniciada inequívocamente en la línea limítrofe entre la corona y la raíz dentarias.

5: Caries radicular. Lesión cariosa iniciada en alguna porción expuesta de la raíz.

6: Otros valores. Este apartado permite incluir situaciones no descritas con anterioridad.

La gravedad o severidad de la caries se evaluó de la siguiente manera ^3^^,^^4^:

A: Esmalte/cemento. Son las lesiones más superficiales e indican que la caries se encuentra situada en la corona, sin superar el espesor del esmalte, o en la raíz, sin superar el espesor del cemento radicular.

B: Dentina. Son las lesiones de gravedad media, en las que la destrucción de tejido dentario alcanza la dentina, pero no la rebasa.

C: Pulpa. Son las lesiones más graves, en las que la destrucción de tejido dentario ha alcanzado la parte vital del diente, lo que afecta vasos y nervios.

Las osamentas objeto del presente estudio, están debidamente clasificadas y ordenadas según el contexto al que pertenecen y forman parte de colecciones osteológicas humanas del Gabinete de Antropología Física de la Dirección Regional de Cultura Cusco. Únicamente se realizó la eliminación del polvo (con pera de aire), pues las osamentas ya habían sido previamente limpiadas por el personal del gabinete.

Todo el proceso de diagnóstico fue realizado por un único observador, quien fue calibrado por una antropóloga experta (EPT). En el caso de la caries, esta fue localizada por inspección visual con lupas de aumento 6X. El diagnóstico de caries se confirmó con la retención del extremo de un explorador dental en el interior de la lesión teniendo en cuenta la superficie dental afectada por la patología y el grado de penetración de esta.

El sexo y la edad, así como la procedencia, fueron tomados de los registros de la institución (estudios previos).

### Análisis de datos

Todos los datos fueron procesados mediante el paquete estadístico IBM SPSS Statistic 26, con el que se realizó un análisis de distribución de frecuencias de las muestras según las variables de sexo, edad y grupo dentario. Cuando fue necesario comparar, se utilizó el análisis de chi-cuadrado. El nivel de confianza fue del 95%.

## RESULTADOS

Se estudió a 39 individuos en la colección de Sacsayhuamán, de los cuales el 25,6% (n = 10) fueron de sexo masculino; el 69,2% (n = 27) de sexo femenino y 5,1% (n = 2), de sexo indeterminado. En el caso de los 49 individuos de la colección de Machupicchu, el 75,5% (n = 37) fueron varones; el 12,2% (n = 6), mujeres; y un 12,2% (n = 6), de sexo indeterminado.

Según grupo etario, las muestras estuvieron distribuidas de la siguiente forma: Sacsayhuamán estuvo conformada por un 2,6% (n = 1) de individuos indeterminados, un 51,3% (n = 20) de adultos jóvenes, un 41% (n = 16) de adultos medios y un 5,1% (n = 2) de adultos mayores; mientras que Machupicchu estuvo integrada por un 14,3% (n = 7) de individuos indeterminados, un 51% (n = 25) de adultos jóvenes, un 32,7% (n = 16) de adultos medios y un 2% (n = 1) de adultos mayores.

El total de piezas dentarias analizadas en el presente estudio fue de 1033, de las cuales 566 correspondieron a individuos de la muestra Sacsayhuamán y 467 a la muestra Machupicchu.

La muestra Sacsayhuamán estuvo formada por un 18,2% (n = 103) de incisivos, un 11,3% (n = 64) de caninos, un 30,9% (n = 175) de premolares y un 39,6% (n = 224) de molares; mientras que la muestra Machupicchu estuvo formada por un 12,8% (n = 60) de incisivos, un 12,4% (n = 58) de caninos, un 31,5% (n = 147) de premolares y un 43,3% (n = 202) de molares.

**Figura 1:**
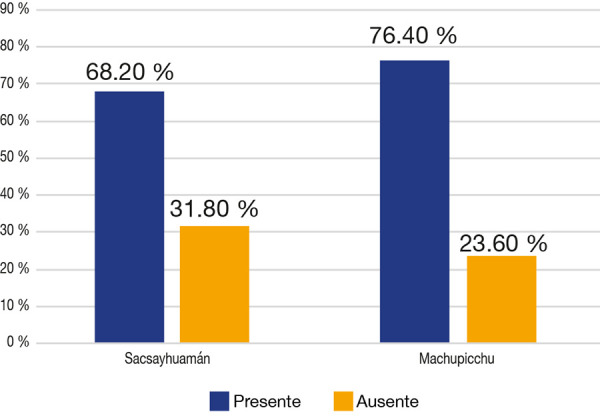
Frecuencia de caries en las muestras de Sacsayhuamán y Machupicchu

En ambas muestras, el mayor porcentaje fueron piezas libres de caries, con valores en Sacsayhuamán del 68,2% (n = 388) y en Machupicchu del 76,4% (n = 357), mientras que las piezas afectadas de caries en Sacsayhuamán fueron un 31,8% (n = 178) y en Machupicchu, un 23,6% (n = 110). Respecto de la caries, existe una diferencia estadísticamente significativa (p < 0,05) (Figura 1).

Chi cuadrado = 7,930 P = 0,005

**Figura 2:**
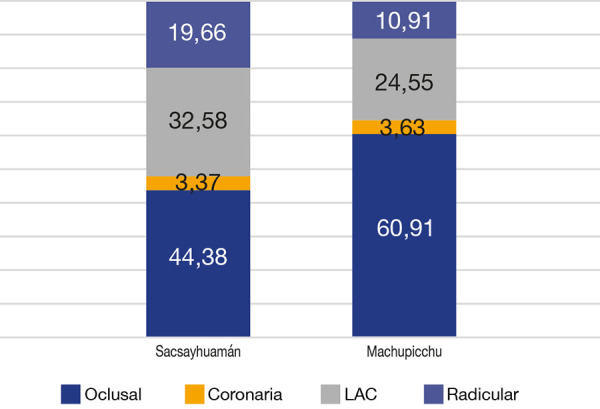
Prevalencia de la caries dental según su localización: Sacsayhuamán versus Machupicchu

En cuanto a las piezas dentarias que presentaron caries según su localización, encontramos, en la muestra Sacsayhuamán, caries oclusal en el 44,38% (n = 79), caries coronaria en el 3,37% (n = 6), caries en la línea amelocementaria (LAC) en el 32,58% (n = 58) y caries radicular en el 19,66% (n = 35); mientras que en la muestra Machupicchu, caries oclusal en el 60,91% (n = 67), caries coronaria en el 3,62% (n = 4), caries en la línea amelocementaria (LAC) en el 24,55% (n = 27) y caries radicular en el 10,91% (n = 12). Respecto de la localización de la caries, existe una diferencia estadísticamente significativa (p < 0,05) (Figura 2).

Chi cuadrado = 8,358 P = 0,039

**Figura 3:**
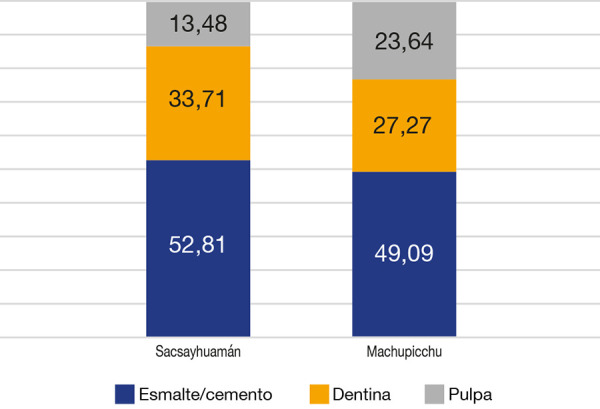
Prevalencia de la caries dental según su severidad: Sacsayhuaman versus Machupicchu

Con relación a las piezas dentarias que presentaron caries según su grado-severidad, encontramos, en la muestra Sacsayhuamán, caries esmalte/cemento en el 52,81% (n = 94), caries de dentina en el 33,71% (n = 60) y caries de afectación pulpar en el 13,14% (n = 24); mientras que en la muestra Machupicchu, caries esmalte/cemento en el 49,09% (n = 54), caries de dentina en el 27,27% (n = 30) y caries de afectación pulpar en el 23,64% (n = 26). Respecto de la severidad de la caries, no existe una diferencia estadísticamente significativa (p > 0,05) (Figura 3).

Chi cuadrado = 5,121 P = 0,077

**Tabla 1:** Prevalencia de la caries dental según localización

Localización de la caries		Muestra		Total
		Sacsayhuamán	Machupicchu	
Caries oclusal	n	79	67	146
	%	44,4%	60,9%	50,7%
Caries coronaria	n	6	4	10
	%	3,4%	3,6%	3,5%
Caries en línea amelocementaria	n	58	27	85
	%	32,6%	24,5%	29,5%
Caries radicular	n	35	12	47
	%	19,7%	10,9%	16,3 %
Total	n	178	110	288
	%	100%	100%	100%

**Tabla 2:** Prevalencia de la caries dental según su grado severidad

Estructura afectada		Muestra		Total
		Sacsayhuamán	Machupicchu	
Esmalte/Cemento	n	94	54	148
	%	52,8%	49,1%	51,4%
Dentina	n	60	30	90
	%	33,7%	27,3%	31,2%
Pulpa	n	24	26	50
	%	13,5%	23,6%	17,40%
Total	n	178	110	288
	%	100%	100%	100%

## DISCUSIÓN

**Figura 4:**
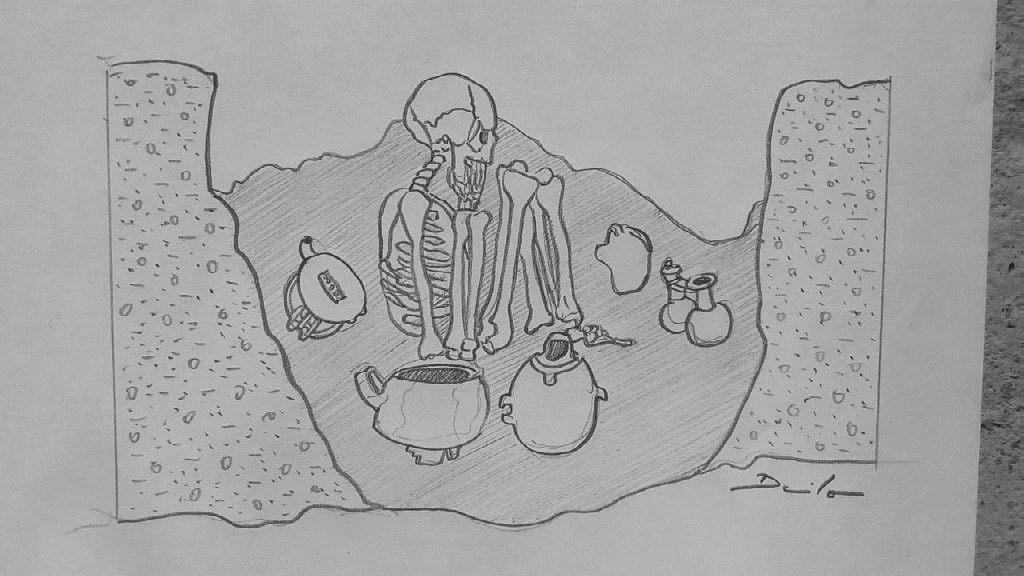
Restos encontrados en entierros similares al contexto

**Figura 5:**
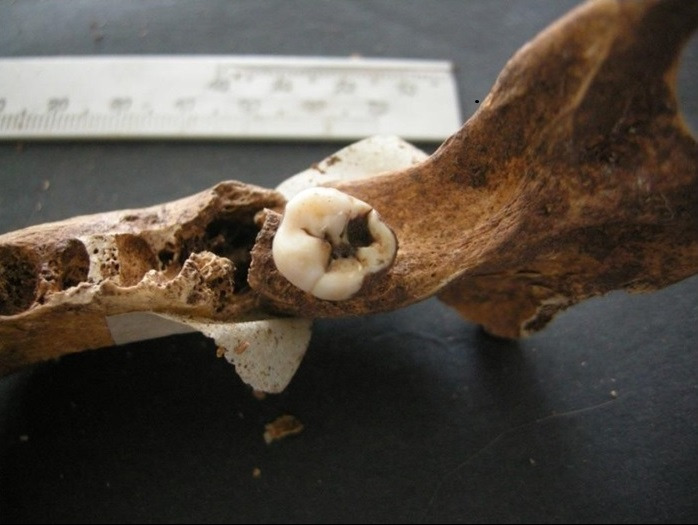
Localización oclusal y afectación dentinaria

**Figura 6:**
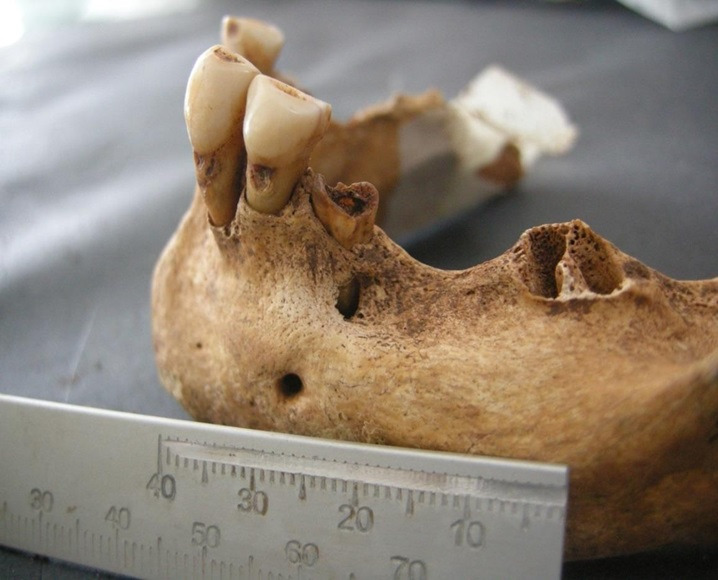
Localización coronaria y afectación pulpar, obsérvese piezas perdidas ante mortem (alveolo reabsorbido)

**Figura 7:**
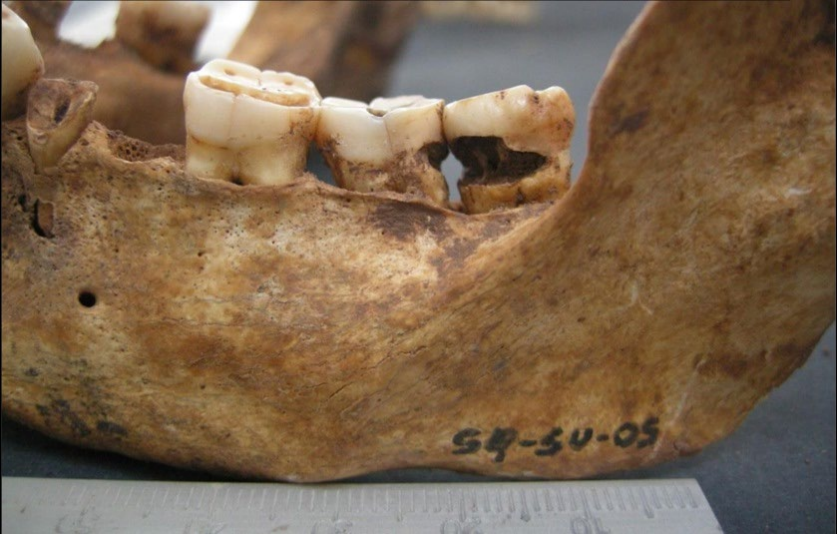
Amplia lesión cariosa de localización en la línea amelocementaria y de afectación pulpar

**Figura 8:**
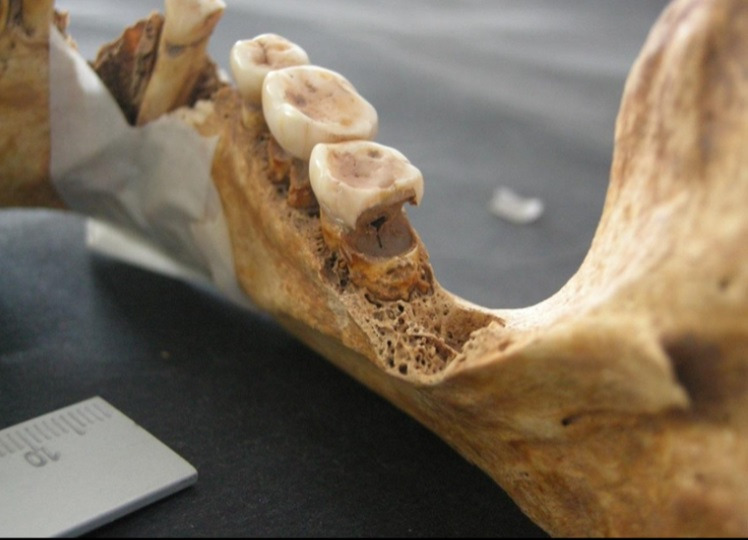
Caries coronaria y radicular que afecto la pulpa dental. Obsérvese el amplio desgaste de las caras oclusales.

**Figura 9:**
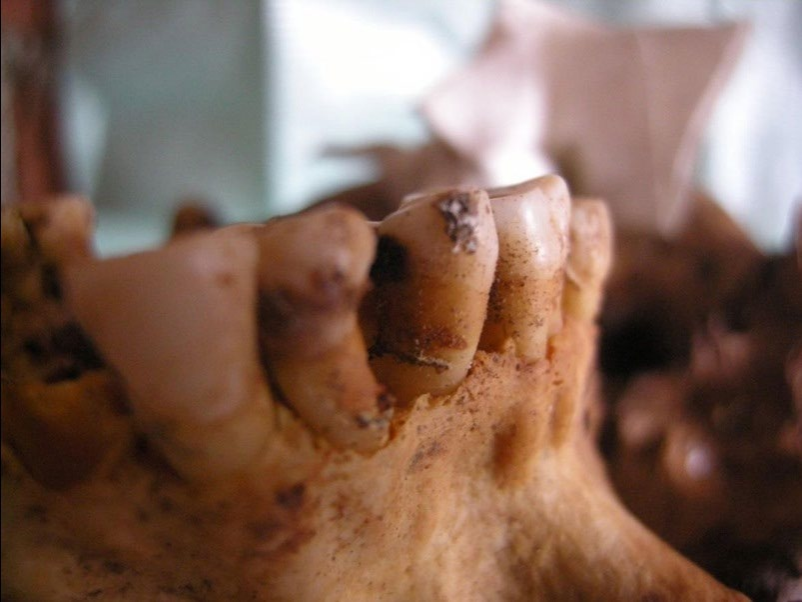
Caries de localización coronaria y que afectó únicamente al esmalte.

**Figura 10:**
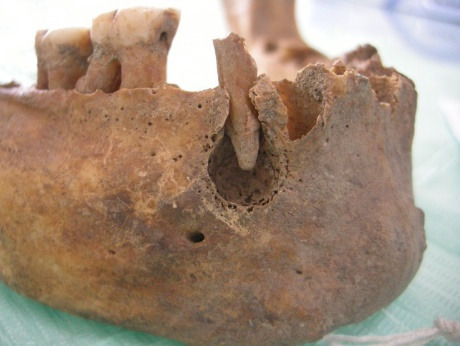
Caries de localización coronaria y afectación pulpar. Obsérvese la amplia lesión periapical con destrucción del hueso.

En este estudio, se analizó la frecuencia, severidad y localización de la caries dental en dos muestras del periodo inca: Sacsayhuamán y Machupicchu. Tras la revisión, se halló que en ambas el número de piezas sanas (sin caries) es superior al de piezas cariadas, con valores mayores al 50% de las piezas estudiadas. (Figuras 4-10).En Sacsayhuamán se obtuvo un 68,6 % de piezas sin caries y un 31,4 % de piezas dentales cariadas, mientras que en Machupicchu observamos que las piezas sin caries son el 76,4 % y las piezas con presencia de caries, el 23,6%. 

Si comparamos este resultado con poblaciones modernas, Alvarado ^13^ encontró una prevalencia de caries del 79,49 % en una muestra conformada por adolescentes de instituciones educativas de Lima (Perú) en 2005, por lo que corroboramos que la caries es más frecuente en sociedades modernas que en las del pasado.

Al comparar los resultados con otros obtenidos en investigaciones previas, podemos ver que, en Argentina, Mansegosa ^14^ encontró una frecuencia del 12,5% de piezas cariadas y el 87,5% de piezas libres de caries en una muestra colonial de Tucumán. En otro estudio, Pezo ^12^ encontró frecuencias de caries que oscilan entre el 40% y el 42% en muestras preíncas de la costa peruana. Por su parte, Tomasto ^15^ halló la siguiente frecuencia de caries en muestras preíncas procedentes de la costa peruana, por periodo: arcaico, 25,7%; paracas, 43,4%, y nazca, 45,8%.

Podemos ver resultados parecidos en muestras de la sierra; sin embargo, si analizamos las muestras de la Costa, esto aumenta significativamente y también lo hace con el tiempo. Por tanto, podemos pensar, como dice Borja ^8^, que antaño “la caries afecta más a la población de la Costa y la Selva que la población del Ande”.

Al comparar una y otra muestra con respecto a la frecuencia de caries, observamos diferencias estadísticamente significativas (p < 0,05), siendo mayor la frecuencia de caries en la muestra Sacsayhuamán, hecho que podríamos explicar porque ese sector pertenecía a la periferia de la capital del imperio (Cusco), donde llegaban alimentos del todo el Tahuantinsuyo, a diferencia de Machupicchu, que se encontraba más lejos, por lo que su acceso a una alimentación más diversificada pudo estar limitado. 

Por otro lado, la mayor frecuencia de caries dental está relacionada con un nivel socioeconómico más bajo ^7^^,^^19^. Por ello, podríamos pensar que la gente que habitó Machupicchu tenía un mayor estatus social (sacerdotes y nobleza), mientras que las personas de la muestra Sacsayhuamán podrían provenir del vulgo del imperio. En tal sentido, estaríamos introduciendo una nueva variable además de la alimentación, que sería el nivel socioeconómico.

Es importante tener en cuenta que hay una disparidad en las muestras con respecto al sexo de los individuos: la muestra de Sacsayhuamán es mayoritariamente femenina, mientras que la de Machu Picchu es mayoritariamente masculina. Esto introduciría otra nueva variable: el sexo, lo que debería ser tomado en consideración en futuras investigaciones. Además, es posible que estos resultados se expliquen por el hecho de que solo estamos examinando las piezas dentales que han sobrevivido, sin conocer el destino de las piezas perdidas, tanto antes como después de la muerte. Las piezas dentales perdidas antes de la muerte podrían haber sido consecuencia de la progresión de la caries, mientras que las piezas perdidas después de la muerte podrían haber mostrado lesiones cariosas de diferentes grados de afectación ^16^. Este hecho constituye, por tanto, una importante limitación para el estudio.

En cuanto a su localización, sin tener en cuenta las piezas libres de caries, podemos ver que existen diferencias significativas entre uno y otro grupo (p < 0,05). En ambas muestras, la caries de localización oclusal fue la que alcanzó mayores valores, seguida por la caries en la línea amelocementaria (LAC), la caries radicular y, finalmente, la caries coronaria. Podemos ver que, si bien los valores mayores corresponden a la caries oclusal, tanto la caries radicular como la de línea amelocementaria mostraron importantes porcentajes, quizás explicados por una enfermedad periodontal previa que expuso estas zonas ^17^. La diferencia estadísticamente significativa (p < 0,05), en el caso de la caries oclusal, la encontramos a favor de la muestra Machupicchu, dado que está asociada a una dieta más cariogénica ^8^. Podríamos pensar que la muestra Machupicchu, al estar en ceja de selva, tuvo un mayor acceso a alimentos ricos en azúcares, como frutas ^(18, 19)^. Si hablamos de las caries en la línea amelocementaria y radicular, encontramos diferencias estadísticamente significativas (p < 0,05), esta vez en favor de la muestra Sacsayhuamán, lo que es atribuible a una dieta más fibrosa, que expuso las porciones radiculares y cervicales para el posterior desarrollo de caries ^17^. Ambas afirmaciones nos indican un mayor consumo de alimentos fermentables en la muestra Machupicchu y una dieta mayoritariamente fibrosa en la muestra Sacsayhuamán, hecho que corresponde a la diferencia en los alimentos consumidos en las regiones Sierra y ceja de selva.

En cuanto a la caries dental, según su severidad, no encontramos diferencias estadísticamente significativas entre uno y otro grupo (p > 0,05); sin embargo, en ambos grupos vemos que la mayor severidad fue la de esmalte/cemento, lo que nos haría pensar que la caries dental afectó solo levemente a ambas muestras, es decir, si hubo caries dental esta fue leve (esmalte/cemento). No obstante, cabe señalar que las poblaciones en estudio alcanzaron una expectativa de vida bastante corta, pues en la categoría adulto mayor se encuentran personas mayores de 45 años, que en nuestras muestras son el grupo etario más pequeño; quizás esta corta esperanza de vida pudo influir en la gravedad de la caries dental, pues lo individuos morían por diferentes razones antes que progrese la caries dental ^8^.

Limitaciones: En nuestras muestras existen más individuos en la muestra Machupicchu que en la muestra Sacsayhuamán, pero hay más piezas dentarias en la muestra Sacsayhuamán que en la muestra Machupicchu. Quizás el estado de conservación entre una y otra muestra debe ser considerado también un factor de sesgo, lo que podría haber influido tanto en la localización como en la severidad de caries dental. Asimismo, podemos ver que existen más piezas posteriores (premolares y molares), que piezas anteriores (incisivos y caninos) en ambas muestras en estudio, esto debido a la facilidad con que las piezas anteriores pueden desprenderse del cráneo seco. Esta circunstancia también pudo influir en los resultados de la presente investigación. En estudios de este tipo, es difícil poder encontrar muestras similares y que cumplan con todos los criterios de comparación, por lo que esta investigación debe ser tomada como una referencia, más que como una representación de las poblaciones estudiadas.

## CONCLUSIONES

- Al comparar las muestras, se encontraron diferencias con respecto a la frecuencia de caries dental y su localización, mas no se encontraron diferencias en cuanto a su severidad.
- En la muestra Sacsayhuamán, las piezas con caries alcanzaron una frecuencia del 31,8% y fueron la caries de localización oclusal y de severidad esmalte/cemento las de mayor frecuencia. La mayoría de piezas dentarias están libres de caries y, si bien su localización mayoritaria es oclusal, su severidad es leve.
- En la muestra Machupicchu, las piezas con caries alcanzaron una frecuencia del 23,6% fueron la caries de localización oclusal y la de severidad esmalte/cemento las de mayor frecuencia. En esta muestra también se aprecian mayoritariamente piezas libres de caries, cuya localización es oclusal y su severidad, leve.
